# Fabrication of Long‐Lasting Superhydrophilic Anti‐Fogging Film Via Rapid and Simple UV Process

**DOI:** 10.1002/advs.202409463

**Published:** 2024-11-08

**Authors:** Jae Hwan Jung, Yu Bin Kang, Chungryong Choi

**Affiliations:** ^1^ Department of Polymer Science and Engineering Kumoh National Institute of Technology 61 Daehak‐ro Gumi Gyeongbuk 39177 Republic of Korea

**Keywords:** anti‐fogging, durability, superhydrophilic polymer, thiol‐ene click, UV‐induced crosslinking

## Abstract

Surface fogging is a common phenomenon that can result in restricted visibility, reduced light absorption, and image distortion. Although both hydrophobic and hydrophilic surfaces are effective in preventing this phenomenon, typical coatings in both have limitations, including low durability and the need for frequent reapplication. To address these issues, a highly durable anti‐fogging film that lasts over five weeks, even under high moisture conditions, while maintaining a promising degree of transparency (> 60%) is developed. A novel statistical random copolymer containing superhydrophilic and photo‐crosslinkable segments that can be simultaneously crosslinked and chemically bonded to various substrates via a simple ultraviolet (UV) irradiation process is synthesized. Notably, the chemical bonding between the anti‐fogging coating and substrate improves not only the durability but also the resistance to external forces and environmental changes. Furthermore, this film is versatile and applicable to diverse substrates, such as car windshields, polymer films, and aluminum foil. The innovative strategy offers a simple, rapid process and durable anti‐fogging performance with broad applications in the automotive industry, optical devices, and building materials.

## Graphical abstract

A newly synthesized superhydrophilic polymer can be crosslinked and form chemical bonds with various substrates simultaneously using sunlight. Surfaces coated with this polymer exhibit rapid water absorption, a low contact angle, and high transparency even in high humidity conditions. Additionally, the chemical bonding between the polymer and the substrate imparts greater durability compared to commercial products.

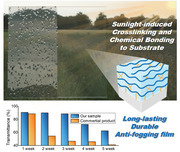

## 1 Introduction

Fogging is a common phenomenon with significant negative impacts such as restricted visibility,^[^ ^1^ ^]^ reduced light absorption in solar cells,^[^ ^2^, ^3^ ^]^ and image distortion in lenses.^[^ ^4^, ^5^, ^6^ ^]^ Hence, it is crucial to develop an effective method to prevent fogging. Fogging occurs when warm humid air contacts a cold surface, and the air temperature drops below the dew point, resulting in the formation of water droplets.^[^ ^7^, ^8^, ^9^, ^10^, ^11^ ^]^ This can be prevented by making a surface either hydrophobic or hydrophilic. Hydrophobic coatings reduce the adhesion of water droplets to the surface and increase their repulsion,^[^ ^12^, ^13^, ^14^ ^]^ and sprays, gels, and wipes are examples of commercially available hydrophobic treatments.^[^ ^15^ ^]^ However, hydrophobic coatings exhibit anti‐fogging effects only when water droplets grow large enough to detach from the surface or when external force (e.g., gravity and wind) is applied to move droplets.^[^ ^16^, ^17^ ^]^ Additionally, these coatings have drawbacks such as low durability and short lifespan because they can be readily removed by physical impacts or environmental changes, necessitating frequent reapplication. By contrast, hydrophilic coatings effectively absorb water droplets when exposed to moisture, thereby forming an even surface.^[^ ^18^, ^19^, ^20^, ^21^, ^22^ ^]^ Thus, many researchers have focused on hydrophilic polymers, including poly(vinyl alcohol) (PVA), as an exemplary material for hydrophilic coatings.^[^ ^23^, ^24^, ^25^, ^26^ ^]^ To enhance the durability of PVA‐based hydrophilic coatings, PVA is primarily crosslinked through a thermal process, which involves relatively complex and time‐consuming processes.^[^ ^27^, ^28^, ^29^ ^]^ Furthermore, even though a polymer film is crosslinked on a substrate, the interaction at the interface between the crosslinked coating and the substrate consists only of physical adhesion (e.g., van der Waals forces and dipole‐dipole interactions). This makes the crosslinked polymer film susceptible to detachment under external forces.^[^ ^27^ ^]^

To address these limitations, simplify the process, and improve the durability of PVA anti‐fogging coating films, Qui and coworkers used photopolymerization to form a hetero‐network of PVA while simultaneously inducing chemical bonding with the substrate using silane chemistry.^[^ ^27^ ^]^ However, typical hydrophilic polymers such as PVA still have limitations because of their slow water absorption and inability to form a perfectly even surface, resulting from relatively high contact angle. To address these issues in PVA‐based anti‐fogging systems, Pester and coworkers introduced a superhydrophilic polymer brush.^[^ ^30^, ^31^, ^32^, ^33^ ^]^ Superhydrophilicity refers to a characteristic that limits the contact angle within 10°, allowing excellent water absorption, and is primarily observed in ionic polymers. Despite the advancements made in these notable studies, a significant reduction in transparency was observed within 1 h of exposure to moisture because of the inevitably low durability resulting from the nanometer‐scale thickness of the polymer brushes.

In this study, a highly durable anti‐fogging film that could last over five weeks was introduced. A newly synthesized superhydrophilic polymer was simultaneously crosslinked and chemically bonded to various substrates via a simple ultraviolet (UV) irradiation process. To accomplish this, a statistical random copolymer consisting of ≈98 mol% superhydrophilic poly[2‐(methacryloyloxy)ethyl trimethylammonium chloride] (PMETAC) and ≈2 mol% crosslinkable poly(allyl methacrylate) (PAMA) was prepared. The synthesized polymer was crosslinked in the presence of a photoinitiator through a thiol‐ene click reaction within 3 s using pentaerythritol tetrakis(3‐mercaptopropionate) (PETMP) as a crosslinker.^[^ ^34^, ^35^, ^36^ ^]^ Simultaneously, the synthesized polymer was chemically bonded to various thiolated (‐SH) surfaces, resulting in long‐lasting performance. Notably, the developed system demonstrated durability and persistence, maintaining a transmittance exceeding 60% over five weeks of exposure to moisture, whereas commercially available products and crosslinked PVA showed a significant decrease in transmittance (< 50%) in the same period. Additionally, the versatility of the synthesized polymer was demonstrated by its application to diverse substrates, such as car windshields, polymer films, and aluminum foil. It is also noteworthy that a low contact angle was preserved even after multiple instances of external physical forces. This strategy promises a simple and rapid process with versatility for application to diverse substrates. Therefore, the proposed anti‐fogging film is expected to have broad applications in various fields, such as the automotive industry, optical devices, and building materials.

## 2 Results and Discussion

### 2.1 Design and Synthesis of Superhydrophilic and Crosslinkable Polymer (PMETAC‐*stat*‐PAMA)

**Figure 1:**
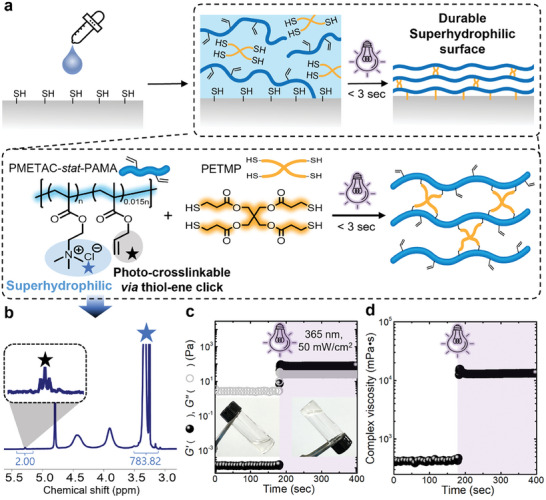
a) Schematic of fabricating durable anti‐fogging film using PMETAC‐*stat*‐PAMA on a thiolated surface via photopolymerization. b) The ^1^H NMR spectrum indicates a PMETAC to PAMA ratio of 100:1.5. c) UV‐rheometer result shows that photo‐crosslinking is completed within 3 s. d) The PMETAC‐*stat*‐PAMA solution can be easily coated due to its appropriate viscosity.

In this study, we propose a strategy for fabricating durable and effective anti‐fogging films by synthesizing a novel superhydrophilic polymer that is simultaneously crosslinked and chemically bonded to a substrate surface via photoinduced thiol‐ene click reaction (**Figure** **1**a). Although an anti‐fogging film can be directly polymerized on a substrate initiated from small molecules (e.g., monomers, initiators, and crosslinkers), it is challenging to quantitatively analyze the effect of each component. In addition, the unreacted small molecules may pose toxicity concerns. To achieve a green and non‐toxic process, the desired statistical random copolymer as a precursor was obtained through free‐radical polymerization, which involved adding [2‐(methacryloyloxy)ethyl]trimethyl‐ammonium chloride solution (METAC) and allyl methacrylate (AMA) monomers in a 100:1 feed ratio in an aqueous solution. The synthesized polymer is a statistical random copolymer consisting mostly of superhydrophilic PMETAC groups based on ammonium chloride and a small number of crosslinkable PAMA groups. Notably, when the feed ratio of the resulting copolymer exceeded 100:2, the viscosity of the resulting polymer in an aqueous solution increased dramatically, making it challenging to achieve a uniform coating. This may be attributed to the phase separation caused by the solubility difference between the hydrophilic PMETAC and hydrophobic PAMA groups. The allyl group in PAMA was introduced because 1) it has lower reactivity compared to methacrylate, and thus does not participate in free radical polymerization; and 2) it undergoes post‐modification through photoinduced thiol‐ene click reaction.^[^ ^37^ ^]^ Meanwhile, when the monomer concentration during polymerization was high and the polymerization time was too long, resulting in high conversion, the allyl group of the AMA monomer also participated in polymerization, leading to undesired crosslinking. Therefore, the monomer concentration and the reaction time were fixed at 10 wt% and 12 h, respectively. As shown in Figure 1b (see also Figure S1, Supporting Information), the synthesized polymer, denoted as PMETAC‐*stat*‐PAMA, was quantitatively analyzed using proton nuclear magnetic resonance (^1^H NMR). The ratio of PMETAC to PAMA was calculated by comparing the integration of the distinct signals from the allyl group at 5.3 ppm and methyl ammonium ≈3.4 ppm. The results revealed that the ratios of PMETAC to PAMA in the feed ratio and the resulting polymers were nearly consistent at 100:1 and 100:1.5, respectively.

The synthesized polymer can be photo‐crosslinked through thiol‐ene click reactions with multifunctional thiols, resulting in gelation (Figure S2, Supporting Information), which enables simpler and faster processes than conventional methods such as thermal crosslinking.^[^ ^38^, ^39^ ^]^ It is noted that curing utilizing UV possesses several benefits: 1) the reaction doesn't require any instruments and can be achieved even with sunlight; 2) in the case of thiol‐ene reaction, it does not produce by‐products; (3) it can improve productivity by removing heating and cooling process and shortening manufacturing time. To optimize the ratio of the crosslinker to the added PMETAC‐*stat*‐PAMA, the storage and loss moduli were monitored after crosslinking with various allyl to thiol ratios in a 10 wt% aqueous solution. The highest crosslinking efficiency was achieved when the allyl‐to‐thiol ratio was 1:0.75, which yielded the highest storage modulus. Therefore, the ratio of the synthesized copolymer to tetra‐thiol crosslinker was fixed accordingly (Figure S3, Supporting Information). By observing moduli changes during photo‐crosslinking under UV irradiation (365 nm, 50 mW cm^−^^2^) with a UV‐rheometer, it was confirmed that crosslinking where the storage and loss moduli are crossed completed within 3 s, indicating gelation (Figure 1c; see also Figure S4a, Supporting Information). Since the thiol‐ene reaction is initiated by light, the reaction temperature does not affect the performance of the film (Figure S4b, Supporting Information). Moreover, the complex viscosity of the prepared solution at a concentration of 10 wt% before UV irradiation was measured to be ≈400 mPa·s, similar to that of castor oil (250–500 mPa·s) (Figure 1d). This viscosity suggests that the solution can be readily used in various coating methods such as bar coating, dip coating, and blade coating.

### 2.2 Influence of Film Thickness on Anti‐Fogging Performance

**Figure 2:**
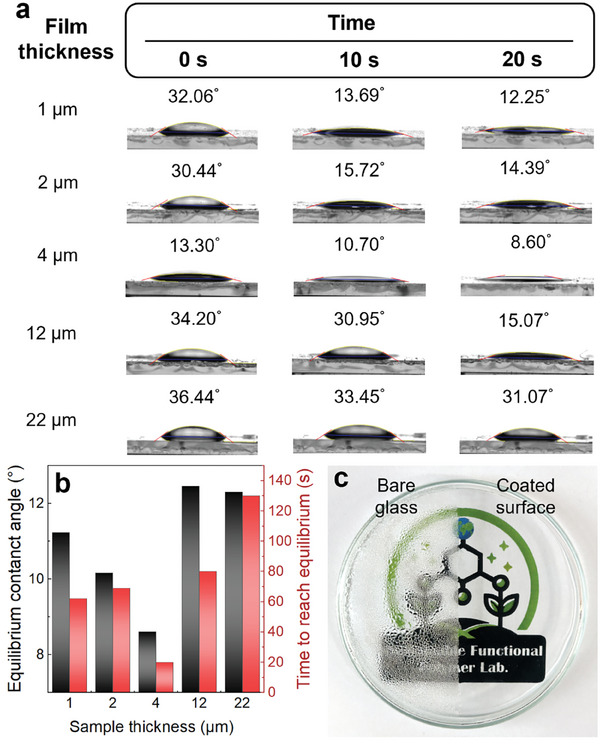
Optimization of film thickness. a) Contact angle measurements for various coating thicknesses (1‐22 µm) over time. b) Time‐dependent contact angle analysis showing 4 µm as optimal thickness. c) Demonstration of anti‐fogging performance: bare glass (left) shows water condensation while coated surface (right) maintains clear visibility.

The parameters determining anti‐fogging performance, including the contact angle and water absorption time, were significantly influenced by the film thickness.^[^ ^40^, ^41^, ^42^ ^]^ To determine the optimal coating thickness for anti‐fogging, crosslinked PMETAC‐*stat*‐PAMA with various thicknesses (1, 2, 4, 12, 22 µm) were prepared on a glass substrate. **Figure** **2**a shows the contact angle changes for the water droplets on the crosslinked PMETAC‐*stat*‐PAMA film over time, up to 20 s. On the thickest 22 µm film, the highest contact angle (36.44°) was observed immediately after contact, with little change in 20 s. In contrast, the thinnest 1 µm film showed a high initial contact angle (32.06°) but decreased significantly to 12.25° in 20 s, demonstrating a faster water absorption compared to the thicker film. The optimized film thickness of 4 µm offered the lowest contact angle of 13.30° immediately after contact as well as the fastest water absorption, resulting in 8.60° in 20 s. For a quantitative comparison, Figure 2b summarizes not only the time taken to reach equilibrium but also the equilibrium contact angle with various thicknesses of the crosslinked PMETAC‐*stat*‐PAMA. Although all coatings showed a decreasing trend in contact angle over time, the lowest contact angle (8.60°) and the shortest time to reach equilibrium (20 s) were exhibited at the 4 µm thickness (See Supporting Information Movies). The superior performance of the 4 µm film compared to thinner films is due to its higher water absorption capacity. On the other hand, as the film thickness increases, the added crosslinker (PETMP) accumulates at the top surface because of its lower surface energy compared to PMETAC‐*stat*‐PAMA. This leads to the formation of gradient crosslinking across the film thickness, as confirmed by EDS analysis (Figure S5, Supporting Information). Thus, the crosslinked PMETAC‐*stat*‐PAMA at 4 µm thickness demonstrated excellent anti‐fogging performance when exposed to moisture, as shown in Figure 2c. While the bare glass substrate exhibited a transmittance of ≈40% when exposed to moisture for 1 min, the glass substrate where the PMETAC‐*stat*‐PAMA is coated and crosslinked maintained a transmittance of ≈90%, which was nearly identical to that of the bare glass substrate (Figure S6, Supporting Information). Additionally, even after the crosslinked PMETAC‐*stat*‐PAMA film reached saturation, the anti‐fogging performance was maintained (Figure S7, Supporting Information). This is because, once saturated, water droplets are no longer absorbed but instead flow off the surface, resulting in an even surface. As a comparison, when crosslinked PVA as a control, a representative hydrophilic polymer, was applied as an anti‐fogging film according to the literature,^[^ ^43^ ^]^ both the contact angle and absorption time were significantly higher than those of the crosslinked PMETAC‐*stat*‐PAMA film, which is consistent with previous report (Figure S8, Supporting Information).^[^ ^44^ ^]^

### 2.3 Effect of Chemical Bonding Between PMETAC‐*stat*‐PAMA and Substrate

**Figure 3:**
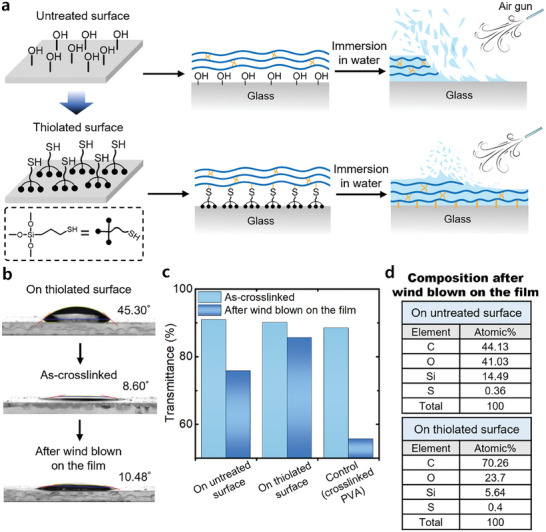
Effect of chemical bonding between anti‐fogging film and substrate. a) Thiolated surfaces form chemical bonds with PMETAC‐*stat*‐PAMA for enhanced durability. b) Contact angle stability after external force application on chemically bonded films. c) Transmittance comparison at 800 nm before and after applying external force (i.g., air blowing), showing superior stability of the chemically bonded coating. d) EDS elemental quantification shows higher carbon content on the thiolated surface than on the unthiolated surface after wind‐blown on the film.

A typical polymer film on a substrate, whether crosslinked or not, interacts with physical forces at the interface between the polymer and substrate, making it susceptible to damage or delamination under external environmental changes.^[^ ^32^, ^45^, ^46^, ^47^, ^48^ ^]^ Conversely, creating chemical bonds at the interface can result in a more durable film capable of withstanding such changes.^[^ ^49^, ^50^, ^51^ ^]^ To clarify the effect of chemical bonds, two samples were prepared. On the bare glass, denoted as the untreated surface, PMETAC‐*stat*‐PAMA was coated and crosslinked, resulting in physical interactions between the surface and the crosslinked polymer (**Figure** **3**a). The other sample was prepared on a thiol‐modified glass with (3‐mercaptopropyl)trimethoxysilane to create a thiolated substrate. Because the synthesized PMETAC‐*stat*‐PAMA polymer contains allyl groups capable of forming a chemical bond with thiol via a thiol‐ene click reaction, it can be chemically grafted onto a thiolated surface. Therefore, UV irradiation enabled PMETAC‐*stat*‐PAMA to simultaneously crosslink with the multifunctional thiol crosslinker (PETMP) and form chemical bonds with the thiolated surface. Thus, the fabrication process is simplified and accelerated compared to the typical thermal crosslinking process. Notably, when crosslinked PMETAC‐*stat*‐PAMA was applied to the untreated surface, it was readily detached by an external force, resulting in an increase in the contact angle from 10.76° to 29.14° (Figure S9, Supporting Information). In contrast, the crosslinked PMETAC‐*stat*‐PAMA chemically bonded to the thiolated substrate exhibited a negligible change in contact angle, from 8.6° to 10.48°, before and after applying an external force. (Figure 3b).

Figure 3c compares the transmittance at a wavelength of 800 nm for the control and our sample before and after applying an external force at room temperature (*i.g*., air blowing at a speed of 14.6 m s^−1^, at an angle of 45°, 15 cm apart from the sample, for 10 s). The PMETAC‐*stat*‐PAMA film chemically bonded to the thiolated substrate showed minimal change in transmittance, whereas the PMETAC‐*stat*‐PAMA film on the untreated substrate exhibited a significant decrease in transmittance from 90% to 75% (Figure S10, Supporting Information). Furthermore, the PVA control sample showed considerably lower transmittance than our sample after an external force was applied, with the transmittance decreasing from 88% to 57% after air was blown onto the sample surface. It is hypothesized that, when the substrate and polymer are chemically bonded, even though the film may be partially delaminated, the PMETAC‐*stat*‐PAMA chemically bonded to the bottommost layer forms a single layer that continues to function as an anti‐fogging film. The influence of chemical bonding was confirmed by analyzing the chemical composition of the crosslinked PMETAC‐*stat*‐PAMA on thiolated and untreated substrates using energy‐dispersive spectroscopy (EDS) after air blowing (Figure 3d; Figure S11, Supporting Information). The results revealed that in the absence of chemical bonding, the carbon content was relatively low at 44%, whereas in the presence of chemical bonding, it was high at 70%. Thus, when the polymer and the substrate are chemically bonded, the higher resistance to external forces prevents the polymer film from completely delaminating, thereby imparting durability. Additionally, to determine whether a single layer of PMETAC‐*stat*‐PAMA can function as an anti‐fogging film, chemically bonded PMETAC‐*stat*‐PAMA to a thiolated surface without a crosslinker were prepared, resulting in only chemical bonding between the polymer and the substrate without crosslinking (Figure S12, Supporting Information). After washing the non‐crosslinked PMETAC‐*stat*‐PAMA polymer film, resulting in a single layer of PMETAC‐*stat*‐PAMA bonded to the thiolated substrate, the contact angle of the monolayer was 10.61°, confirming that it still exhibited superhydrophilic characteristics. Overall, while chemical crosslinking of superhydrophilic polymers is a promising technique for imparting anti‐fogging properties, it often results in delamination from the substrate due to insufficient interfacial adhesion. To address this, introducing a chemical bond between the substrate and the hydrophilic film is a key strategy for enhancing interfacial adhesion, thereby improving durability against external forces, while maintaining hydrophilic properties. Although anti‐fogging films based on hydrophilic polymers tend to have low contamination resistance due to their high surface energy, our experiments did not show any significant decrease in transmittance due to this issue (Figure S13, Supporting Information). Therefore, the crosslinked PMETAC‐*stat*‐PAMA film could be a promising candidate for a durable and high‐performing anti‐fogging film.

### 2.4 Anti‐fogging Duration of Crosslinked PMETAC‐*stat*‐PAMA Coating

**Figure 4:**
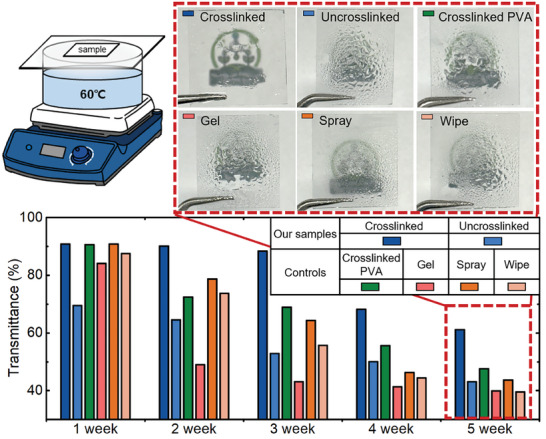
Transmittance comparison of various samples at 800 nm wavelength to verify persistence to high moisture conditions at 60 °C for five weeks. The crosslinked PMETAC‐*stat*‐PAMA on the thiolated substrate demonstrates excellent moisture resistance, maintaining over 60% transmittance for five weeks.

To compare the persistence of anti‐fogging performance under high moisture conditions, samples including commercially available products (gel, spray, and wipe mainly containing sorbitol, *α*‐Isotridecyl‐*ω*‐hydroxypoly(oxy‐1,2‐ethanediyl), perfluorooctanoic acid as the small molecule components for anti‐fogging, respectively), PVA, and our PMETAC‐*stat*‐PAMA, were prepared and exposed to moisture at 60 °C for up to 5 weeks. As shown in **Figure** **4** (See also Figures S14 and S15, Supporting Information), the experiment was set up such that heated water vapor repeatedly condensed on the surface of the samples and fell back into the water bath. The transmittance was measured at one‐week intervals.

After one week of moisture exposure, most samples, except for uncrosslinked PMETAC‐*stat*‐PAMA, maintained a high transmittance of over 90%. The uncrosslinked PMETAC‐*stat*‐PAMA exhibited a dramatic decrease in transmittance owing to polymer dissolution in the condensed water droplets. In commercially available gel, spray, and wipe products, which are surfactant‐based, a distinct decline in anti‐fogging performance was observed after two weeks because the surfactants were washed away by condensed water. In contrast, the transmittance of the crosslinked PVA was relatively longer but decreased to below 50% by the fifth week. Remarkably, the PMETAC‐*stat*‐PAMA crosslinked on the thiolated substrates outperformed the others, maintaining over 60% transmittance until the fifth week and demonstrating the highest persistence. As discussed above, it can be postulated that while crosslinked PVA, which physically adheres to the substrate, results in reduced durability, PMETAC‐*stat*‐PAMA achieves enhanced persistence due to chemically bonding with the substrate. After deterioration under high moisture conditions, the crosslinked PMETAC‐*stat*‐PAMA can be easily renewed by reapplying and curing the polymer, as it crosslinks with the remaining layer of the deteriorated film (Figure S16, Supporting Information). Consequently, the crosslinked PMETAC‐*stat*‐PAMA coating exhibited superior durability and moisture resistance compared to the other controls, which was attributed to both polymer crosslinking and chemical covalent bonding with the substrate.

### 2.5 Versatility of PMETAC‐*stat*‐PAMA Coating on Various Surfaces

**Figure 5:**
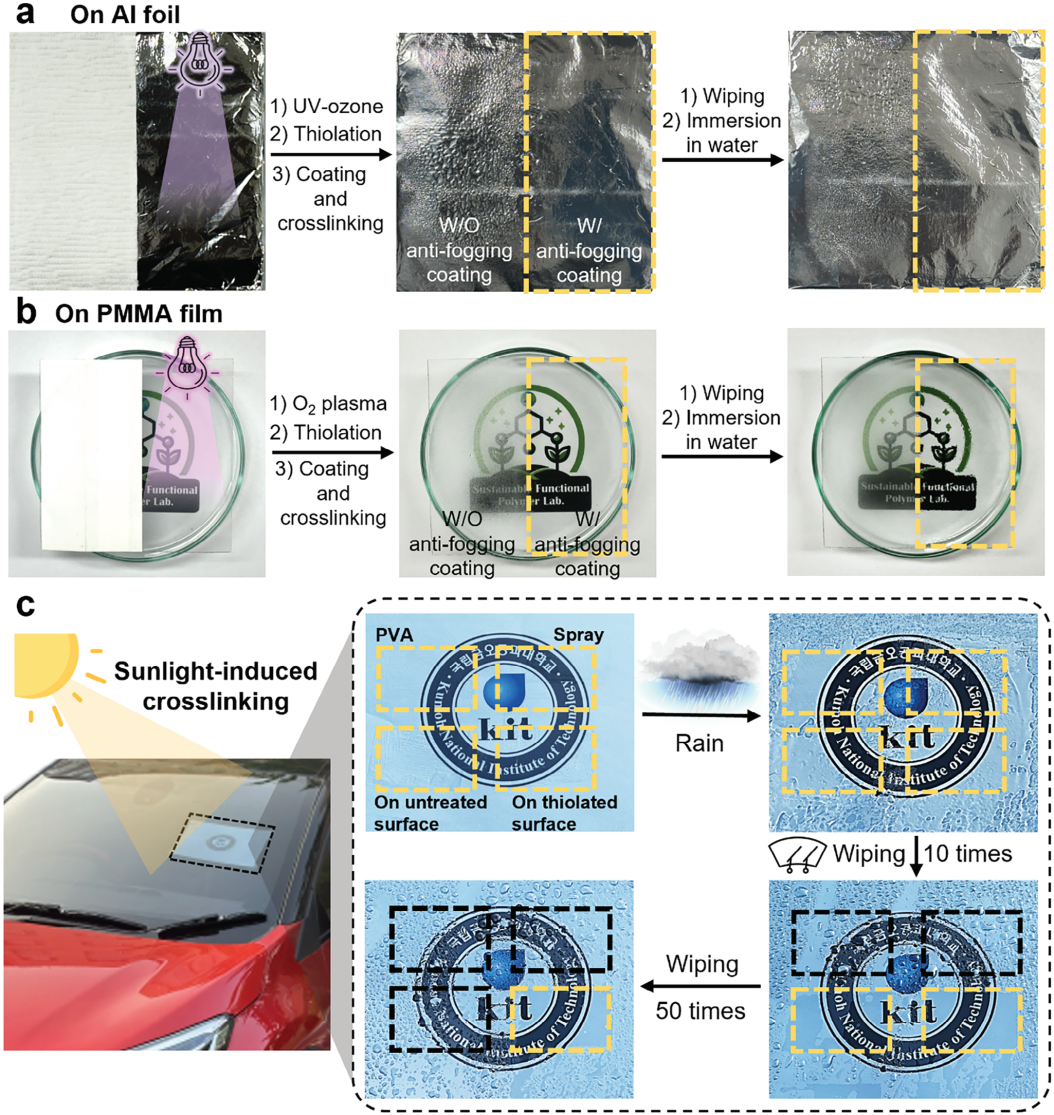
Versatility of PMETAC‐*stat*‐PAMA on various surfaces. Anti‐fogging performance on a) aluminum foil and b) PMMA film. Both maintained anti‐fogging effect after manual wiping. c) Durability comparison after 50 wiping cycles: the cured PMETAC‐*stat*‐PAMA on thiolated windshield via sunlight maintains anti‐fogging effect, while unthiolated surface and other coatings (PVA, spray) lose effectiveness. Yellow/block‐dotted boxes indicate effective/ineffective anti‐fogging areas.

The synthesized PMETAC‐*stat*‐PAMA polymer forms chemical bonds on thiol‐treated substrates regardless of the substrate type. To demonstrate its versatility, PMETAC‐*stat*‐PAMA was applied to thiolated aluminum foil and poly(methyl methacrylate) (PMMA) polymer films (**Figure** **5**a,b). The aluminum foil and PMMA films were spatially masked and treated with UV‐ozone and O_2_ plasma, respectively, to form hydroxyl groups on their surfaces, followed by surface modification with 3‐mercaptopropyltrimethoxysilane. As shown in Figure 5a,b, the area on which PMETAC‐*stat*‐PAMA was coated and crosslinked (right side) exhibited an anti‐fogging effect. Water droplets were uniformly spread on the thiolated aluminum and PMMA substrates, resulting in a clear appearance even after exposure to moisture. It was also noted that PMETAC‐*stat*‐PAMA maintained its anti‐fogging performance even after repeated manual wiping owing to its chemical bonding with the substrate. Figure 5c shows the potential application of the PMETAC‐*stat*‐PAMA anti‐fogging film in car windshields. Car windshields are usually exposed to UV radiation and naturally contain numerous hydroxyl groups on their surfaces, allowing for easy thiolation without special treatments such as UV‐ozone or O_2_ plasma treatments. To verify the anti‐fogging performance and durability of the PMETAC‐*stat*‐PAMA film on a car windshield, coatings were spatially fabricated using PVA, commercial spray, and crosslinked PMETAC‐*stat*‐PAMA on both unthiolated and thiolated windshield areas. Initially, all four coatings exhibited anti‐fogging properties. However, after being wiped with a car wiper ten times, the PVA and spray coatings completely lost their anti‐fogging characteristics. Furthermore, after 50 repetitions of wiping with a car wiper, the PMETAC‐*stat*‐PAMA crosslinked on the untreated surface no longer displayed anti‐fogging properties, whereas the polymer crosslinked on the thiolated surface maintained its anti‐fogging characteristics. These results confirmed that the synthesized PMETAC‐*stat*‐PAMA polymer can be utilized as a durable anti‐fogging polymer on various surfaces. With its ability to perform consistently under various conditions (e.g., air blown onto surfaces, and surfaces wiped and washed), the synthesized polymer demonstrates potential for practical applications in diverse environments.

## 3 Conclusion

A novel polymer, PMETAC‐*stat*‐PAMA, was successfully synthesized, incorporating both superhydrophilic and crosslinkable groups. The photo‐crosslinkable allyl groups in the synthesized polymer facilitated simple and straightforward fabrication of durable anti‐fogging films. Additionally, the polymer exhibited an exceptionally low contact angle (< 10°) and rapid water absorption, imparting excellent anti‐fogging performance. The chemical bonding between the polymer and substrate significantly enhanced the resistance to external forces and ensured stability under extended moisture exposure. Unlike commercially available products that lose their anti‐fogging properties within three weeks in humid environments, the synthesized PMETAC‐*stat*‐PAMA, when crosslinked on thiolated substrates, maintained over 60% transmittance for more than five weeks. This technique is versatile and can be applied to various substrates including glass, metals, and polymers. When applied to automotive windshields, PMETAC‐*stat*‐PAMA can form long‐lasting anti‐fogging films by utilizing sunlight, eliminating the need for specialized equipment. Therefore, the PMETAC‐*stat*‐PAMA polymer has exceptional potential as an anti‐fogging film. This chemistry can be extended to diverse applications such as hydrogels, adhesives, and coatings.

## Conflict of Interest

The authors declare no conflict of interest.

## Supporting information



Supporting Information

Supplemental Movie 1

Supplemental Movie 2

Supplemental Movie 3

Supplemental Movie 4

Supplemental Movie 5

## Data Availability

Research data are not shared.
