# Supplementary material for: Fabrication of Long‐Lasting Superhydrophilic Anti‐Fogging Film Via Rapid and Simple UV Process
Source: Adv Sci (Weinh). 2024 Nov 8;12(1):2409463. doi: 10.1002/advs.202409463 (PMC11714152; doi:10.1002/advs.202409463)
Supplement: Supplementary file 1 — Supporting Information [file ADVS-12-2409463-s005.pdf]

ADVANCED SCIENCE
Open Access

Supporting Information

for *Adv. Sci.*, DOI 10.1002/advs.202409463

Fabrication of Long-Lasting Superhydrophilic Anti-Fogging Film Via Rapid and Simple UV Process

*Jae Hwan Jung, Yu Bin Kang and Chungryong Choi**

Supporting information

Fabrication of Long-lasting Superhydrophilic Anti-Fogging Film via Rapid and Simple UV Process

*Jae Hwan Jung, Yu Bin Kang, Chungryong Choi**

Department of Polymer Science and Engineering, Kumoh National Institute of Technology, 61 Daehak-ro, Gumi, Gyeongbuk 39177, Republic of Korea
Email: crchoi@kumoh.ac.kr

Experiments

Materials and Instruments: All chemicals and solvents were used as received, without further purification. [2-(methacryloyloxy)ethyl]trimethyl-ammonium chloride solution (METAC) (408107, 75 wt% in H_2O) was purchased from Sigma-Aldrich (USA). Allyl methacrylate (AMA) (234931, = 98%) and lithium phenyl-2,4,6-trimethylbenzo-ylphosphinate (LAP) (900889, ≥ 95%) were purchased from Sigma-Aldrich. (3-mercaptopropyl)trimethoxysilane (475617, = 95%) was purchased from Sigma-Aldrich. Ammonium peroxodisulfate (APS) (A2098, > 99%), PETMP (P0886, > 90%), and maleic acid (M0006, > 99%) were purchased from TCI. PVA500 (6559-1405), methanol (5558-4404, ≥ 99.8%) and, acetone (≥ 99.6%) were purchased from DAEJUNG (Korea). For crosslinking, UV light was provided using a Thorlabs-DC2200. The anti-fog wipe, gel, and spray products were purchased from Blue Gold (Korea), JYS&TECH (Korea), and Bikemart (Korea), respectively.

The UV-ozone treatment was conducted using an AC-6 UV/ozone cleaner (AHTECH LTS, Korea). Proton nuclear magnetic resonance (^{1}H NMR) spectra were measured using a BRUKER-AVANCE III 400 spectrometer with the deuterated methanol. Field-emission scanning electron microscopy (FE-SEM) images were obtained using a Tescan MAIA 3 LM instrument operating at 10 kV. EDS data for the SEM software were obtained using the Aztec Energy Advanced package. The modulus and viscosity measurements were performed using an Anton Paar Korea Rheometer at 1 Hz, 365 nm, and 3% for 30 min. UV-vis spectrophotometry measurements were performed using a Shimadzu UV-1900 spectrophotometer. The measurements were conducted on coated glass slides in the wavelength range of 400-800 nm. Water contact angle was measured using an SEO Model-PHX300 automated goniometer/tensiometer. The film thickness was measured by ellipsometry.

Synthesis of PTMAC-stat-PAMA: METAC (10 g, 1 eq.) was dissolved along with the initiator APS (0.08 g, 0.01 eq.) in deionized water (100 ml). AMA (0.046 g, 0.01 eq.) was dissolved in a minimal volume of methanol (0.125 ml). Then, the mixture of the prepared solutions was purged under nitrogen atmosphere, and stirred overnight at 70 °C. To remove unreacted monomers, the product was precipitated in acetone three times. Finally, the product was completely dried in a high vacuum oven at 60 °C for a day.

Preparation of Thiolated Surface: The substrates, including glass slides and aluminum foil, were treated with UV-ozone for 45 min to form hydroxyl surfaces. Poly(methyl methacrylate) substrate was exposed to oxygen plasma (FEMTO SCIENCE Instrumentation) at 100 sccm and 250 W for 2 min. The prepared substrates were chemically modified with thiols (-SH) to form a self-assembled monolayer. The detailed method is as follows: (3-mercaptopropyl)trimethoxysilane (2 wt %) was added to a mixed solvent of water and ethanol at a ratio of 99:1. Then, this solution was poured onto the glass slide surface on a hot plate set at 60 °C, ensuring that the entire surface was covered. The slides were then dried in an oven at 60 °C for 2 h. After drying, the glass surfaces were cleaned with ethanol and dried in vacuo.

Fabrication of Anti-fogging Film: PMETAC-*stat*-PAMA (0.1 g) and LAP (0.001 g) were dissolved in 0.75 mg/mL of deionized water. The crosslinking agent was dissolved in a minimal amount of acetone in another vial. The two prepared solutions were mixed so that the ratio of allyl-to-thiol becomes 1:0.75 and coated on the thiolated substrate with thicknesses of 30, 90, 150, and 200 µm using a wet film coater. The coated polymer was crosslinked by being exposed to UV irradiation (365 nm, 50 mW/cm^2), followed by drying.

Preparation of PVA-based Anti-fogging Films: PVA curing conducted according to the literature.[1] Briefly, PVA and maleic acid aqueous solution was prepared at 90 °C. The solution was cast onto glass substrates in the form of a film. The film was dried at ambient temperature and subsequently cured at 120 °C.

Durability Test: Anti-fogging film was applied to both untreated and thiolated surfaces, which were subsequently immersed in water for 1 h. Subsequently, the air was blown onto the coated surfaces using an air gun set at a constant intensity where an air gun with blowing speed of 14.6 m/s is positioned 1 cm above the surface at a 45° angle.

Anti-fogging Test: The anti-fogging performance was evaluated by exposing the film-coated substrates to a humidifier for one minute. Following exposure, the transmittance was measured across a wavelength range of 400-800 nm.

Supplemental Figures

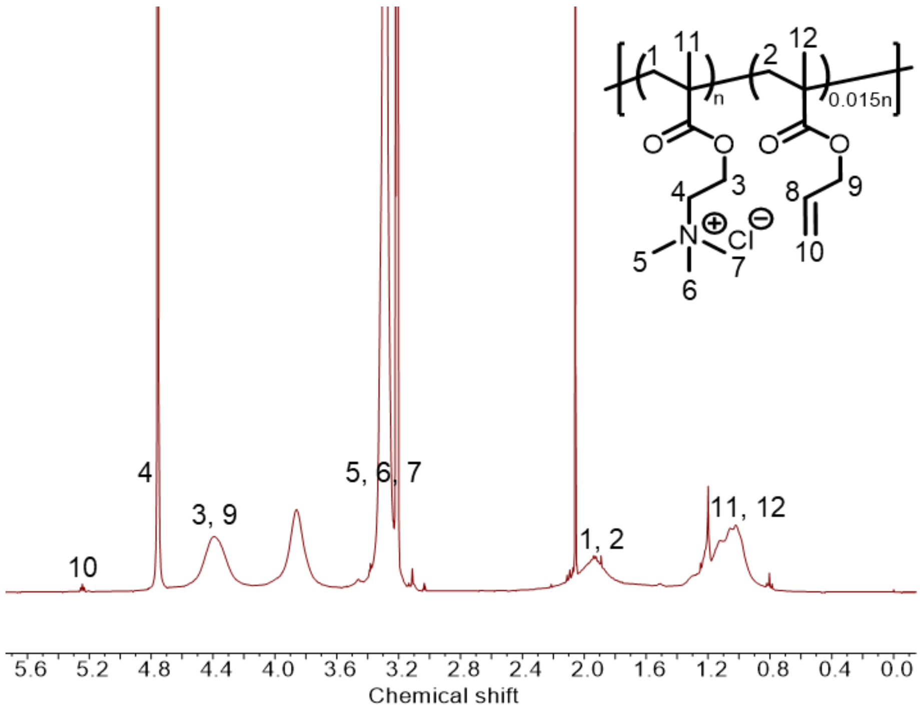

Figure S1. ^{1}H NMR spectrum of the PMETAC-*stat*-PAMA.

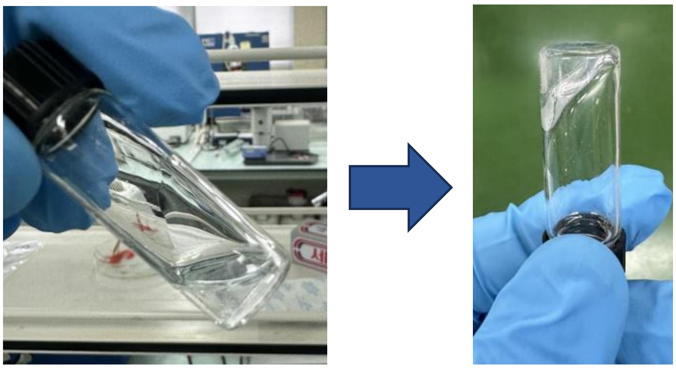

Figure S2. Photo-polymerization via thiol-ene click reaction results in rapid and simple curing with a wavelength of 365 nm light.

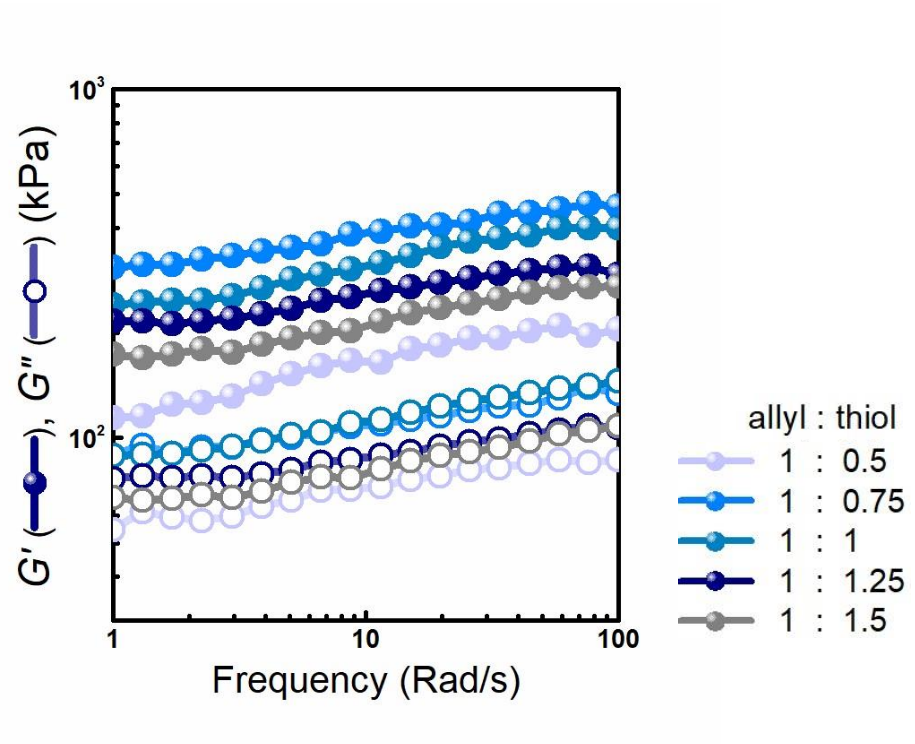

Figure S3. Frequency sweep of the crosslinked PMETAC-*stat*-PAMA in 10 wt% of aqueous solution with various allyl to thiol ratio.

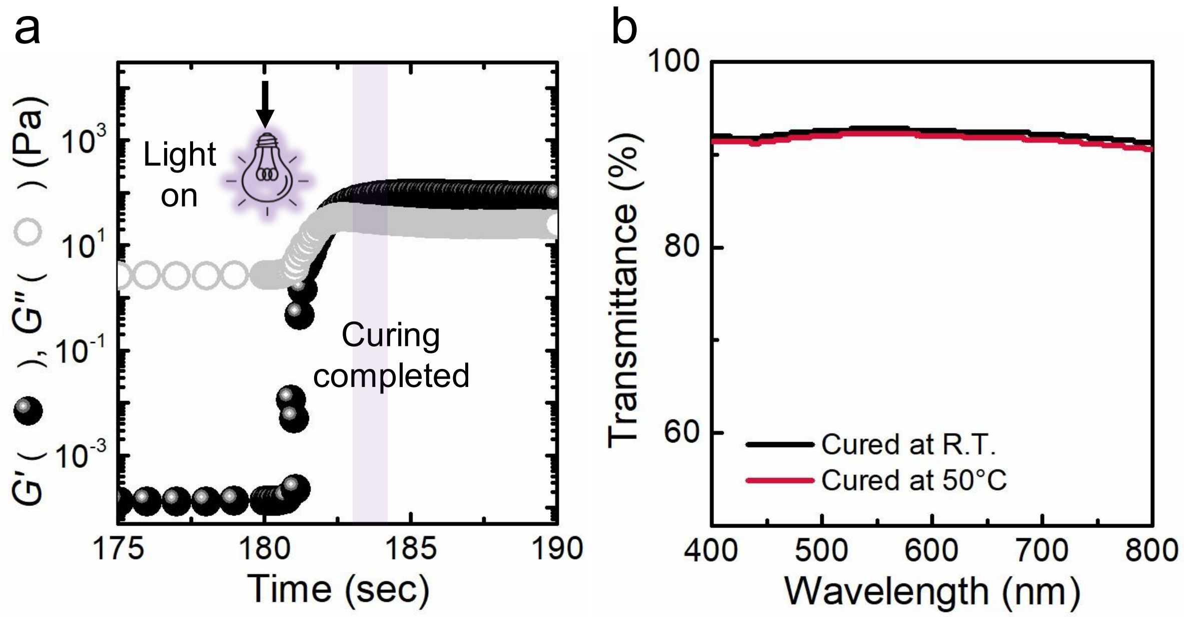

Figure S4. (a) Enlarged curing profile from Figure 1c, showing that curing was completed within 3 seconds. (b) There is no change in the anti-fogging performance of PMETAC-*stat*-PAMA films cured at different temperatures.

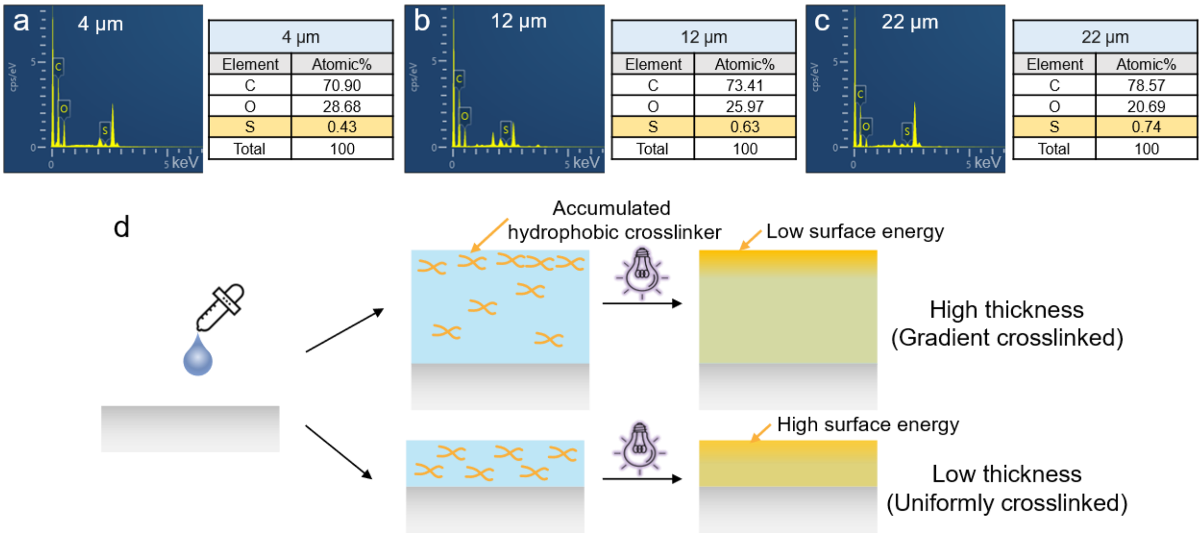

Figure S5. Atomic ratio of crosslinked PMETAC-*stat*-PAMA films with thicknesses of (a) 4 µm, (b) 12 µm, and (c) 22 µm at the top surface, illustrating an increase in sulfur content with greater film thickness. (d) Proposed hypothesis for the higher contact angles and longer absorption times observed in thicker films. This behavior may result from the accumulation of crosslinker in thicker films during the coating and crosslinking processes, driven by surface energy differences between the polymer and crosslinker. Consequently, thicker films exhibit gradient crosslinking and reduced surface energy at the top surface.

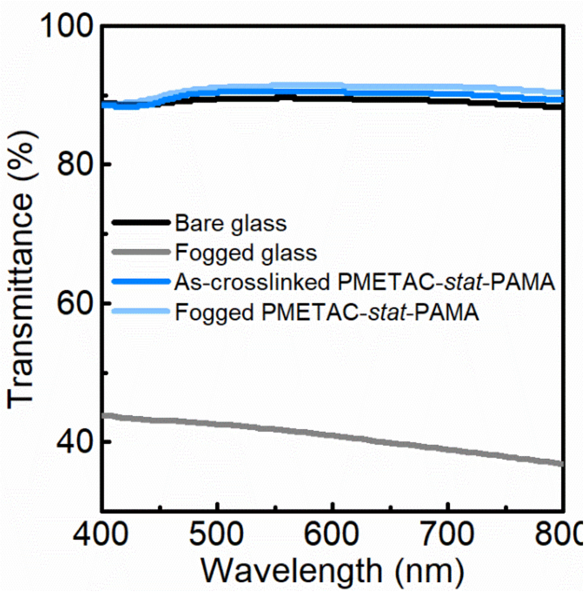

Figure S6. The comparison of transmittance of bare, PMETAC-*stat*-PAMA coated glass.

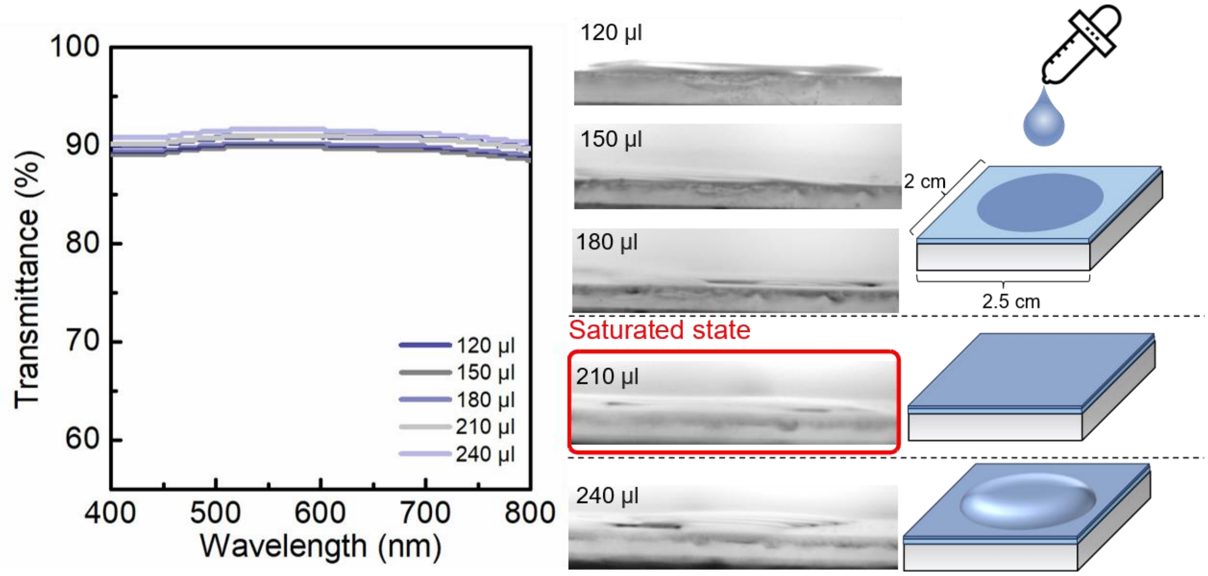

Figure S7. Transmittance and contact angle measurements as a function of the added water volume. When the water volume exceeded 210 μl, the crosslinked PMETAC-*stat*-PAMA film reached saturation, and beyond this point, water droplets became visible on the surface. However, even after reaching saturation, no significant changes were observed in transmittance.

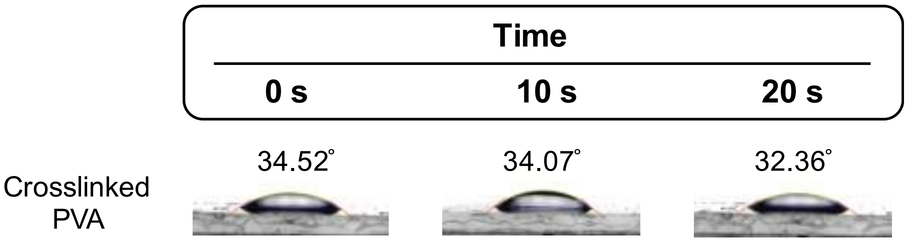

Figure S8. Time-dependent contact angle measurements of crosslinked PVA up to 20 seconds.

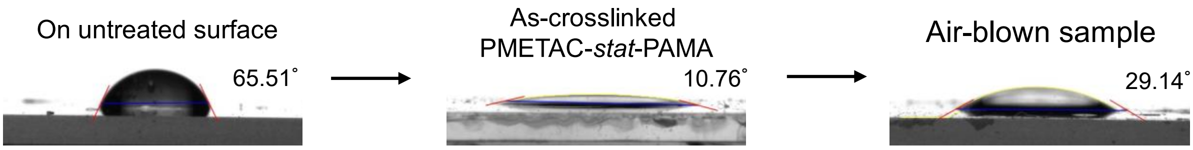

Figure S9. Untreated glass substrate can't form chemical bonds with PMETAC-*stat*-PAMA, decreasing durability and resistance to environmental changes.

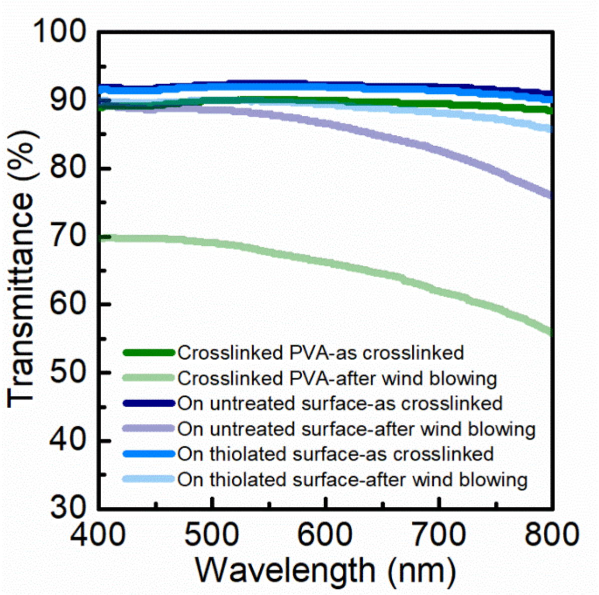

Figure S10. Transmittance changes of the crosslinked PMETAC-*stat*-PAMA on untreated and thiolated surface and crosslinked PVA before and after applying the external force.

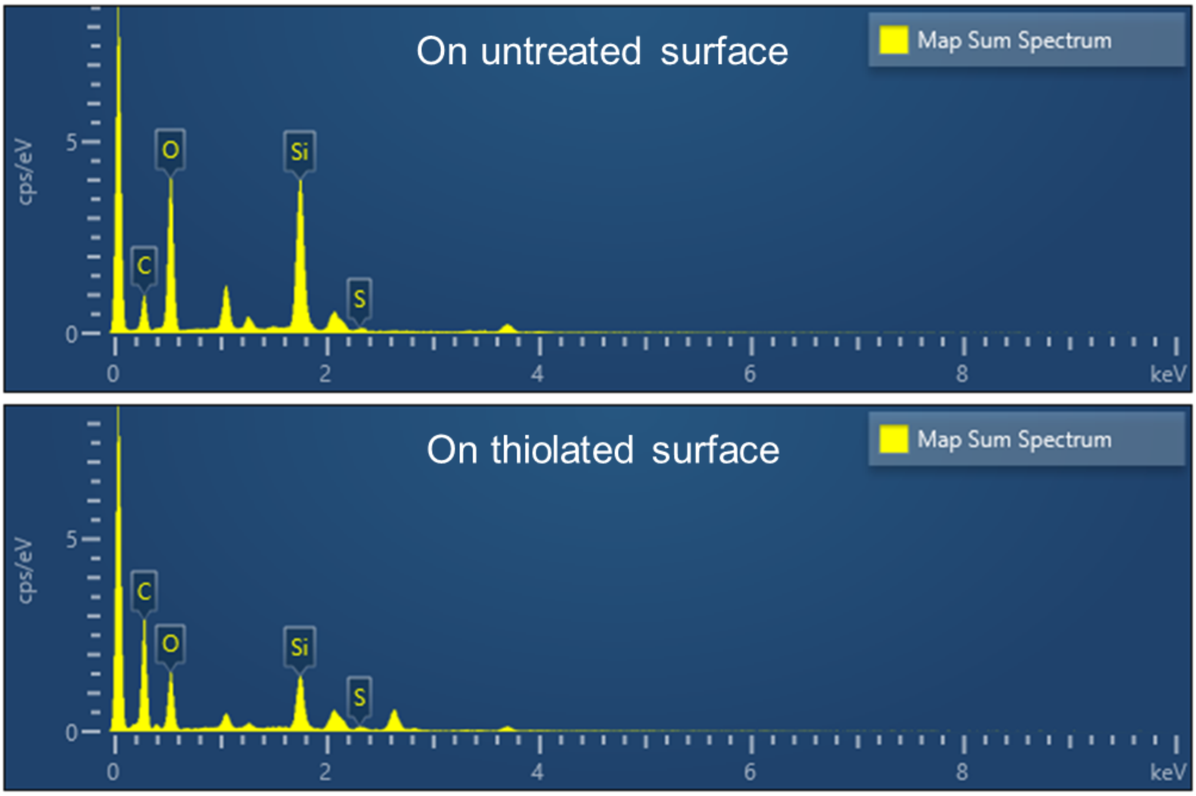

Figure S11. EDS spectra of the crosslinked PMETAC-*stat*-PAMA coating on untreated and thiolated surface after wind blown on the film

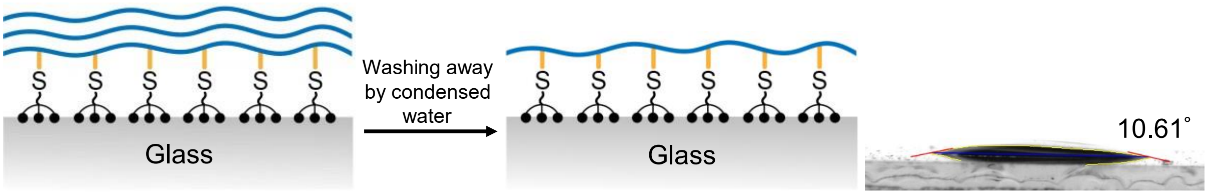

Figure S12. Schematic and contact angle of PMETAC-*stat*-PAMA single layer chemically bonded to the thiolated substrate. This sample was prepared by coating PMETAC-*stat*-PAMA in the absence of a crosslinker followed by rinsing the uncrosslinked PMETAC-*stat*-PAMA.

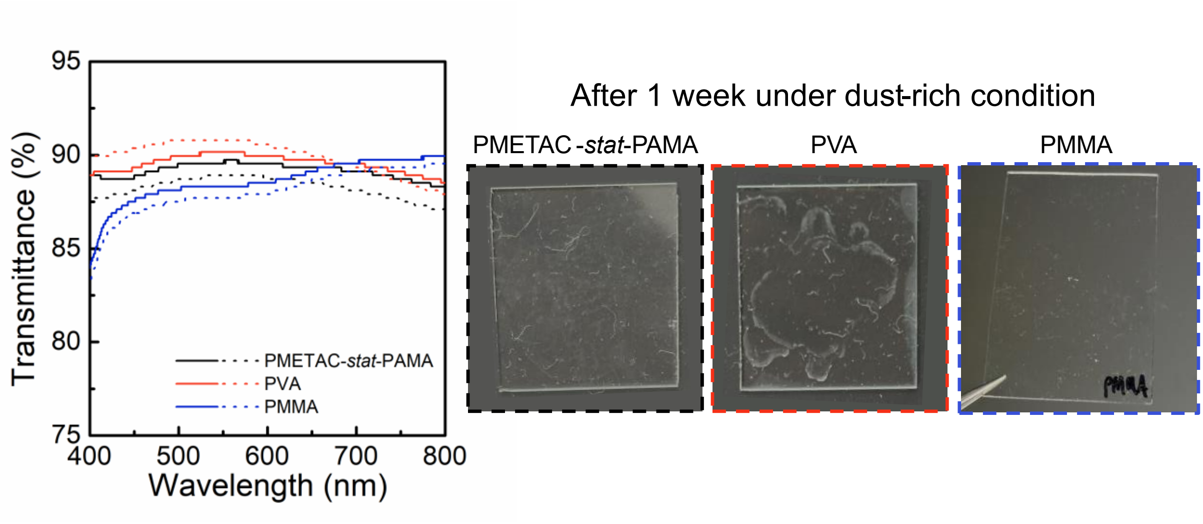

Figure S13. Transmittance changes of PMETAC-*stat*-PAMA, PVA, and PMMA films after exposure to dust-rich conditions for 1 week (solid and dash lines represent before and after exposure, respectively). Although anti-fogging films based on hydrophilic polymers generally have lower contamination resistance due to their high surface energy, no significant changes were observed in our experiment.

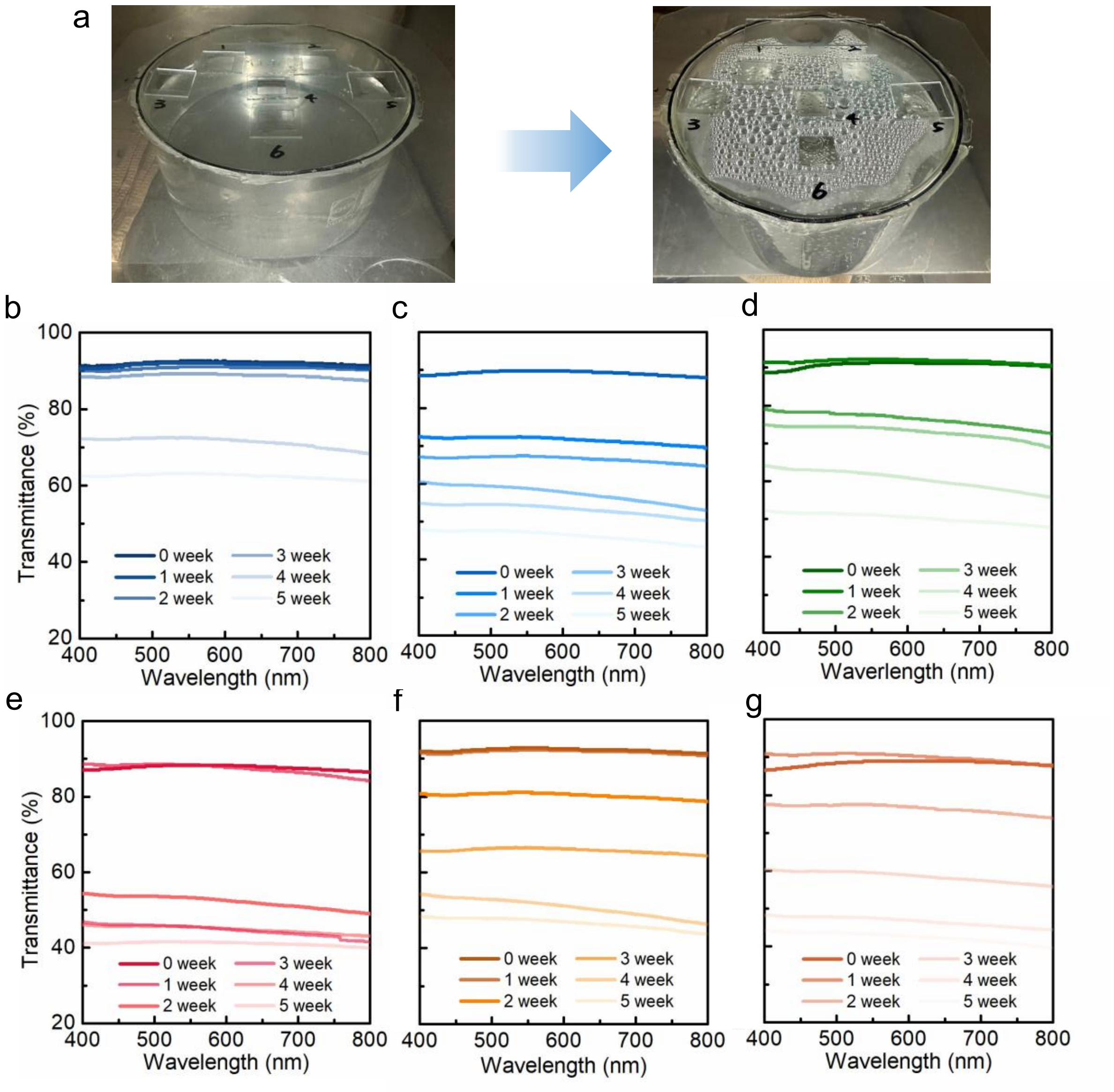

Figure S14. (a) Experiment setup for testing moisture resistance of various anti-fogging coatings**.** Transmittance changes of the (b) crosslinked PMETAC-*stat*-PAMA, (c) uncrosslinked PMETAC-*stat*-PAMA, (d) crosslinked PVA, and commercial anti-fogging (e) gel, (f) spray, (g) wipe for five weeks under high moisture condition. Commercial gel, spray, and wipe mainly contain sorbitol, α-Isotridecyl-ω-hydroxypoly(oxy-1,2-ethanediyl), perfluorooctanoic acid as the small molecule components for anti-fogging, respectively.

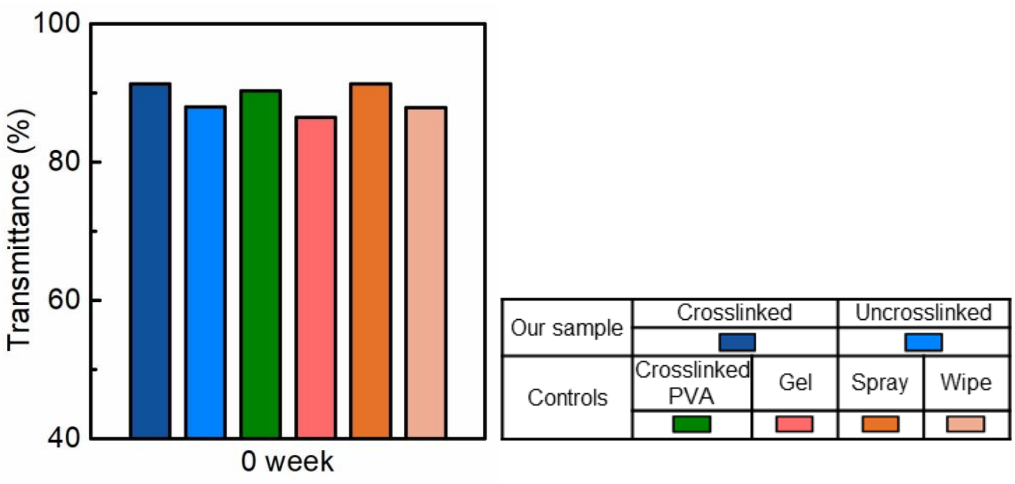

Figure S15. Transmittance comparison at 800 nm wavelength of the fogged PMETAC-*stat*-PAMA, crosslinked PVA, commercial gel, spray, wipe as prepared.

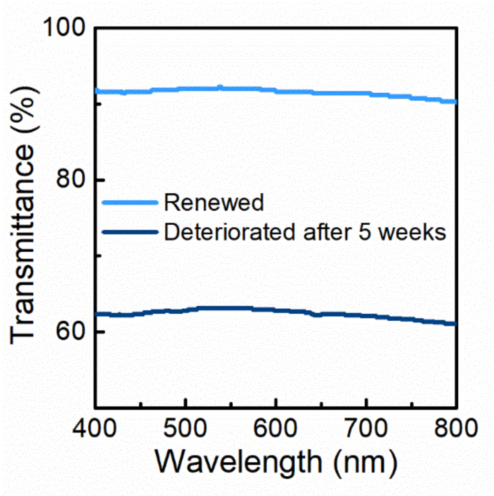

Figure S16. Comparison of the transmittance of crosslinked PMETAC-*stat*-PAMA after exposure to high moisture conditions and the renewed PMETAC-*stat*-PAMA. The renewed sample was prepared by reapplying PMETAC-*stat*-PAMA onto the deteriorated PMETAC-*stat*-PAMA film.

Reference

[1] J. M. Gohil, A. Bhattacharya, P. Ray, *J. Polym. Res.* **2006**, *13* (2), 161-169.
